# The Relationship Between Dietary Inflammatory Index and Intermediate‐to‐High‐Risk Cardiovascular‐Kidney‐Metabolic Syndrome and All‐Cause Mortality: The National Health and Nutrition Examination Survey 1999–2018: A Cross‐Sectional and Prospective Cohort Study

**DOI:** 10.1002/hsr2.71786

**Published:** 2026-02-26

**Authors:** Honglin Li, Mu Lin, Yane Yang, Liyan Wang, Yao Wang, Jiayu Lu, Meiju Li, Yanfei Zhao, Sining Chen, Chenxuan Gao, Chuanwei Zhao

**Affiliations:** ^1^ The Second People's Hospital of Baoshan City Baoshan Yunnan Province China

**Keywords:** cardiovascular–kidney–metabolic syndrome, dietary inflammatory index, mortality, NHANES

## Abstract

**Background:**

The dietary inflammatory index (DII) score quantifies the inflammatory potential of an individual's diet and may be associated with cardiovascular–kidney–metabolic syndrome (CKM) and mortality risk. However, evidence on the relationship between DII and intermediate‐to‐high‐risk cardiovascular–kidney–metabolic syndrome (IH–CKM) is limited.

**Objective:**

This study aimed to evaluate the association between DII and IH–CKM and to explore the relationship between DII and all‐cause mortality risk in individuals with and without IH–CKM.

**Methods:**

We conducted a cross‐sectional and cohort analysis using data from the 1999–2018 National Health and Nutrition Examination Survey (NHANES). Weighted logistic regression models were used to examine associations between DII and IH–CKM, while weighted Cox proportional hazards models estimated hazard ratios (HRs) for all‐cause mortality. Restricted cubic spline analyses were employed to explore linear or nonlinear relationships.

**Results:**

Among 38,732 participants, 30,421 had IH–CKM. Higher DII scores were positively associated with IH–CKM, with a fully adjusted odds ratio (OR) of 1.44 for the highest versus lowest quartile (95% CI: 1.27–1.63, *p* < 0.001). Regarding all‐cause mortality risk, each one‐unit increase in DII (range: −5.281 to 5.795) was associated with an adjusted HR of 1.04 (95% CI: 1.02–1.06, *p* < 0.001) in IH–CKM participants and 1.15 (95% CI: 1.06–1.26, *p* = 0.001) in non‐IH–CKM individuals.

**Conclusion:**

Higher DII scores were positively associated with an increased risk of IH–CKM. In addition, elevated DII was linked to higher all‐cause mortality in both individuals with and without IH–CKM.

**Abbreviations**
ADA: American Diabetes Association
AHA: American Heart Association
BMI: body mass index
CI: confidence interval
CKD: chronic kidney disease
CKD‐EPI: Chronic Kidney Disease Epidemiology Collaboration
CKM: cardiovascular‐kidney‐metabolic
CRP: C‐reactive protein
DBP: diastolic blood pressure
DII: dietary inflammatory index
eGFR: estimated glomerular filtration rate
FPG: fasting plasma glucose
HDL‐C: high‐density lipoprotein cholesterol
HR: hazard ratio
IH–CKM: intermediate‐to‐high‐risk cardiovascular‐kidney‐metabolic syndrome
IL‐10: interleukin‐10
IL‐1β: interleukin‐1 beta
IL‐4: interleukin‐4
IL‐6: interleukin‐6
LDL‐C: low‐density lipoprotein cholesterol
MET: metabolic equivalent tasks
NHANES: National Health and Nutrition Examination Survey
OR: odds ratio
PIR: poverty income ratio
RCS: restricted cubic spline
SBP: systolic blood pressure
SII: systemic immune‐inflammation index
TNF‐α: tumor necrosis factor‐alpha

## 1 Introduction

Chronic, low‐grade inflammation plays a pivotal role in initiating and accelerating the interconnected dysfunction of the cardiovascular, renal, and metabolic systems—collectively referred to as cardiovascular–kidney–metabolic (CKM) syndrome [1]. This multifaceted condition, which increasingly burdens global health systems, is marked by overlapping risk profiles and escalating prevalence across diverse populations [2]. In 2023, the American Heart Association (AHA) underscored the urgency of a unified clinical framework to identify and address CKM, advocating lifestyle‐oriented interventions such as dietary optimization to curb its advancement [3].

One emerging tool for evaluating the proinflammatory nature of diet is the dietary inflammatory index (DII), developed through systematic literature review and later refined to reflect population‐level inflammatory responses to dietary intake [4, 5, 6]. Elevated DII scores, indicative of a proinflammatory dietary pattern, have been consistently linked with higher incidence of cardiometabolic disorders and impaired renal outcomes [7]. Emerging evidence suggests that dietary inflammatory potential may influence CKM progression through the gut microbiota–endotoxin axis. Diets with high inflammatory scores have been shown to alter gut microbial composition, increase intestinal permeability, and facilitate the translocation of lipopolysaccharides (LPS) into the circulation [8, 9]. Elevated circulating LPS triggers systemic low‐grade inflammation, promotes endothelial dysfunction, accelerates renal fibrosis, and contributes to metabolic dysregulation—all of which are central mechanisms underlying CKM development [9, 10]. These pathways provide a plausible biological explanation for the observed associations between pro‐inflammatory dietary patterns and adverse cardiometabolic outcomes. Mechanistically, such diets can exacerbate oxidative stress, disrupt lipid metabolism, promote insulin resistance, and impair endothelial function—pathways intimately tied to the development of CKM syndrome [11]. Complementary evidence has demonstrated that systemic markers of inflammation, including the systemic immune‐inflammation index (SII), are positively correlated with CKM severity, reinforcing the centrality of immune‐metabolic interactions in its pathogenesis [12]. The convergence of cardiovascular, metabolic, and renal impairments suggests a shared inflammatory foundation that may be modifiable through nutritional intervention [13].

While existing research supports the role of inflammation in CKM, there is a paucity of large‐scale population‐based studies directly examining how DII influences both the likelihood of Intermediate‐to‐high‐risk cardiovascular–kidney–metabolic syndrome (IH‐CKM) and associated mortality risk. To address this gap, we investigated the association between DII scores and IH‐CKM status and further explored the relationship between dietary inflammation and all‐cause mortality across CKM subgroups, leveraging data from the National Health and Nutrition Examination Survey (NHANES) spanning 1999 to 2018.

## 2 Materials and Methods

### 2.1 Study Design and Participants

This study utilized data from the NHANES, a cross‐sectional survey program that adopts a multistage probability sampling approach to reflect the health and nutritional status of the U.S. civilian, noninstitutionalized population. NHANES collects detailed information through interviews, dietary recalls, clinical measurements, and laboratory evaluations [14]. The NHANES protocol was approved by the National Center for Health Statistics (NCHS) Research Ethics Review Board, and written informed consent was obtained from all participants during the original survey. Because NHANES datasets are publicly available and fully de‐identified, the present secondary analysis was exempt from additional institutional review board approval and informed consent requirements.

**Figure 1:**
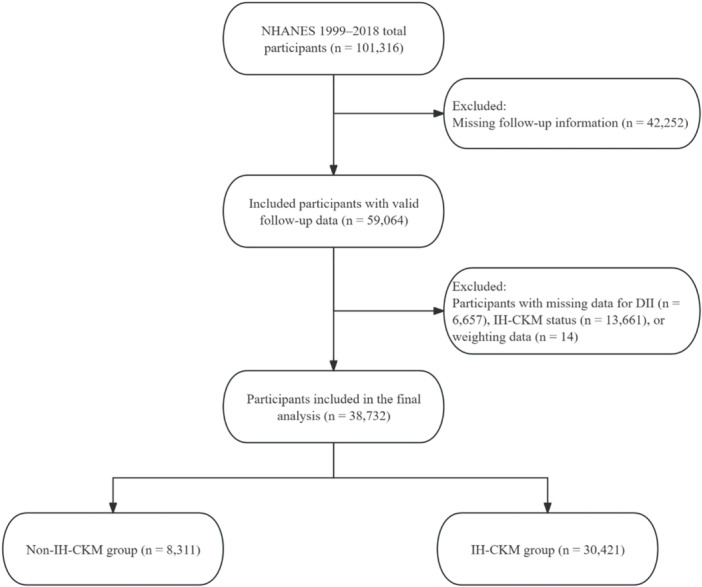
Flowchart of participant selection and inclusion process.

We extracted data spanning from 1999 to 2018. The IH–CKM was defined as CKM stages 2 to 4. CKM stage 2 was defined as the presence of early cardiometabolic or renal abnormalities, including at least one established metabolic risk factor (hypertension, diabetes mellitus, hypertriglyceridemia ≥ 135 mg/dL, or metabolic syndrome) or CKD. CKM stage 3 was defined by elevated predicted cardiovascular risk based on the validated PREVENT risk model, in the absence of clinically overt cardiovascular disease. CKM stage 4 was defined as established cardiovascular disease, including self‐reported coronary heart disease, myocardial infarction, heart failure, or stroke [15]. Hypertension was identified by systolic blood pressure (SBP) ≥ 140 mmHg, diastolic blood pressure (DBP) ≥ 90 mmHg, current use of antihypertensive medications, or self‐reported physician diagnosis [16]. Metabolic syndrome was defined by fulfilling three or more of the following: (1) waist circumference (WC) ≥ 102 cm (men) or ≥ 88 cm (women); (2) triglyceride (TG) level ≥ 150 mg/dL; (3) reduced high‐density lipoprotein cholesterol (HDL‐C): < 40 mg/dL (men) or < 50 mg/dL (women); (4) fasting plasma glucose (FPG) ≥ 110 mg/dL; and (5) blood pressure ≥ 130/85 mmHg [17]. The diagnosis of diabetes followed ADA criteria, which included self‐reported diagnosis, use of glucose‐lowering medications, fasting glucose ≥ 126 mg/dL, or HbA1c ≥ 6.5% [18]. CKD was determined based on an eGFR < 60 mL/min/1.73 m² or a urine albumin‐to‐creatinine ratio > 30 mg/g, using the CKD‐EPI equation published in 2009 [19, 20]. CKM stage 3 classification included individuals at very high risk of CKD progression (G4 or G5 per KDIGO) or those with a predicted 10‐year risk of cardiovascular disease (CVD) ≥ 20%, estimated using the AHA's PREVENT model. Due to NHANES limitations, surrogate cardiovascular biomarkers (e.g., NT‐proBNP, hs‐troponin) and imaging data (e.g., coronary artery calcium, echocardiography) were not available for identifying subclinical CVD. To account for top‐coding of age, individuals aged ≥ 80 were assigned an age of 79 to avoid risk underestimation. CKM stage 4 was defined based on affirmative self‐reported histories of clinically evident CVD, including myocardial infarction, heart failure, coronary heart disease, or stroke, as recorded by standardized NHANES interviews. Diagnoses such as atrial fibrillation or peripheral artery disease were not considered due to unavailable data. We excluded individuals lacking DII data, CKM staging information, survey weights, or mortality follow‐up records. The final analytic cohort is detailed in Figure 1.

### 2.2 Dietary Inflammatory Index

The DII is a standardized scoring tool developed to quantify the inflammatory potential of an individual's diet. In this analysis, the DII score was computed based on the intake of 28 specific dietary components: total energy, macronutrients (carbohydrates, protein, total fat), alcohol, dietary fiber, cholesterol, saturated fat, monounsaturated fatty acids (MUFAs), polyunsaturated fatty acids (PUFAs), omega‐3 and omega‐6 fatty acids, as well as a range of micronutrients including vitamins A, B1 (thiamin), B2 (riboflavin), B3 (niacin), B6, B12, C, D, E, beta‐carotene, folate, and minerals such as iron, magnesium, zinc, selenium, and caffeine [21, 22]. Intake estimates were derived from NHANES 24‐h dietary recall data collected using the Automated Multiple‐Pass Method. Each nutrient or food component was assigned an inflammatory weight based on its documented associations with circulating biomarkers—such as proinflammatory mediators (IL‐1β, IL‐6, TNF‐α, CRP) and anti‐inflammatory markers (IL‐4, IL‐10) [21]. Component scores were standardized to global reference intake values, converted to centered percentiles, multiplied by literature‐derived inflammatory effect scores, and summed to generate an overall DII score. Higher DII values indicate a dietary pattern inclined to promote inflammation, whereas lower or negative scores suggest an anti‐inflammatory dietary profile. The inflammatory effect scores and reference values were derived from a global literature‐based database used in the original DII methodology [23]. When calculated using approximately 25–30 components, the DII typically ranges from −5.5 (strongly anti‐inflammatory) to +5.5 (strongly proinflammatory) [24]. The DII has been validated in prior epidemiologic studies [25, 26].

### 2.3 Follow‐Up and Endpoint

Vital status for each participant was ascertained by probabilistic linkage to the National Death Index Public Access files, with follow‐up censored on December 31, 2019 [24]. The primary endpoint was all‐cause mortality. Mean observation time was 124 ± 2 months for individuals without IH–CKM and 113 ± 1 months for those classified as IH–CKM.

### 2.4 Demographic Characteristics and Other Covariates

Race and ethnicity were categorized as Mexican American, non‐Hispanic Black, non‐Hispanic White, or other, based on NHANES classification standards. Educational level was divided into three groups: less than high school, high school graduate or GED equivalent, and college attendance or higher. Marital status was grouped as married/living with partner, never married, or widowed/divorced/separated. Socioeconomic status was assessed using the poverty income ratio (PIR), which adjusts household income for family size and inflation relative to the federal poverty line. Smoking history was self‐reported and classified as never, former, or current use. Alcohol consumption was dichotomized into active and nonactive use. Physical activity was evaluated using weekly metabolic equivalent task (MET) scores derived from activities such as walking, recreational exercise, and cycling. Information on smoking, alcohol intake, physical activity, hypertension, diabetes, coronary heart disease, heart failure, and stroke was collected through standardized self‐report questionnaires. Anthropometric parameters, including body weight, height, and WC were measured at mobile examination centers following standardized NHANES protocols. Body mass index (BMI) was calculated as weight in kilograms divided by height in meters squared. Laboratory biomarkers assessed included FPG, glycated hemoglobin (HbA1c), serum albumin, creatinine, TG, total cholesterol, low‐density lipoprotein cholesterol (LDL‐C), high‐density lipoprotein cholesterol (HDL‐C), along with BMI and WC.

### 2.5 Statistical Analysis

Given the complex, multistage sampling strategy of the NHANES survey, all statistical procedures incorporated appropriate sampling weights to ensure national representativeness and to adjust for differential selection probabilities and survey nonresponse [27]. Specifically, data from 1999 to 2002 were weighted using “WTDR4YR,” whereas data from 2003 to 2018 applied the “WTDR2YR” weight. Detailed documentation regarding NHANES sampling methodology and analytic guidance is publicly available (https://wwwn.cdc.gov/nchs/nhanes/analyticguidelines.aspx). To evaluate the inflammatory potential of diet, DII scores were divided into four quartiles: Q1 (< 0.35, reference), Q2 (0.35–1.84), Q3 (1.85–3.00), and Q4 (≥ 3.01). These quartile cutoffs were determined based on the weighted distribution of DII in the analytic sample, representing a gradient from relatively low to high dietary inflammatory burden and facilitating interpretability and comparison with prior NHANES‐based studies.

All analyses incorporated NHANES sampling weights, strata, and primary sampling units to account for the complex multistage survey design. Participants were stratified according to IH–CKM status, and baseline characteristics were compared across groups. Continuous variables were summarized as weighted means with standard deviations (SDs), and between‐group differences were assessed using survey‐weighted linear regression. Categorical variables were described as weighted frequencies and proportions, and compared using Rao–Scott adjusted chi‐square tests, as recommended for complex survey data. To evaluate the association between DII and IH–CKM, survey‐weighted logistic regression models were fitted to estimate odds ratios (ORs) with 95% confidence intervals (CIs). Three prespecified models were constructed: an unadjusted model; a model adjusted for age, sex, and race/ethnicity; and a fully adjusted model incorporating education level, marital status, income, smoking status, alcohol consumption, and physical activity. All covariates were selected a priori based on established clinical relevance to cardiometabolic risk and were assessed for multicollinearity prior to model entry. Restricted cubic splines (RCS) is a flexible regression technique used to model potential nonlinear relationships. Potential nonlinear dose–response patterns were examined using RCS with knots placed at the 5th, 50th, and 95th percentiles of the DII distribution. For all‐cause mortality, Cox proportional hazards models were applied, and proportional hazards assumptions were evaluated using Schoenfeld residuals. Hazard ratios (HRs) with 95% CIs were reported for DII as both a continuous variable and quartile categories (Q1 as reference). Survival differences across quartiles were visualized using Kaplan–Meier curves and compared using the log‐rank test. Multiple imputation using chained equations (MICE) is an iterative procedure that imputes missing values based on conditional models, missing covariate data were handled through MICE. Given the complex survey design of NHANES and the relatively low proportion of missing data, multiple imputation was selected as the primary strategy to minimize bias and preserve statistical power. Therefore, a complete‐case analysis was not performed, as it could introduce selection bias under a missing‐at‐random assumption. Predictive mean matching (PMM) is a semi‐parametric imputation method that preserves the distribution of observed data. Five imputed datasets were generated using PMM, and pooled estimates were obtained following Rubin's rules [28]. These quartile cutoffs were determined based on the weighted distribution of DII in the analytic sample, representing a gradient from relatively low to high dietary inflammatory burden and facilitating interpretability and comparison with prior NHANES‐based studies. All categorical variables were defined as factor variables prior to imputation to ensure appropriate modeling behavior. Prespecified subgroup analyses were conducted to examine potential effect modification by sex, age, race/ethnicity, smoking, and alcohol consumption. Interaction terms were evaluated in fully adjusted models. All tests were two‐sided, and a significance level of 0.05 was used for hypothesis evaluation. Analyses were performed using R software (version 4.3.2). Statistical analyses followed current reporting standards, including principles outlined in SAMPL guidelines. All definitions and subgroup categories were prespecified before analysis.

## 3 Results

### 3.1 Baseline Characteristics of the Study Participants

Based on the baseline characteristics in Table 1, 38,732 participants were analyzed, with 8311 in the non‐IH–CKM group and 30,421 in the IH–CKM group. Overall, missingness across covariates was low to moderate, with no variable exhibiting more than approximately 10% missing data. The participants with IH–CKM were older, had a slightly greater proportion of males, and displayed lower educational attainment, with a greater percentage having education below high school. In terms of lifestyle factors, individuals with IH–CKM were more often former smokers, less frequently active alcohol users, and had lower total METs per week for physical activity. Compared with their non‐IH–CKM counterparts, the IH–CKM group presented elevated levels of FPG, HbA1c, serum creatinine, TG, and total cholesterol, along with reduced HDL‐C levels. The IH–CKM group also presented a significantly greater prevalence of hypertension, diabetes, coronary heart disease, congestive heart failure, stroke, CKD, and metabolic syndrome. Additionally, the all‐cause mortality rate was notably greater in the IH–CKM group than in the non‐IH–CKM group. These findings indicate that individuals with IH–CKM experience more adverse health conditions and face a higher mortality risk than those without IH–CKM.

**Table 1:** Baseline characteristics of participants stratified by the presence or absence of IH–CKM.

Characteristic	Overall (*N* = 38,732)	Non‐IH–CKM (*N* = 8311)	IH–CKM (*N* = 30,421)	*p* value
Age, years	49.57 ± 0.20	38.20 ± 0.29	52.90 ± 0.20	< 0.001
Sex (*n*, %)				0.02
Female	19,894 (51.59)	4342 (53.36)	15,552 (51.07)	
Male	18,838 (48.41)	3969 (46.64)	14,869 (48.93)	
Race (*n*, %)				0.06
Mexican American	6930 (7.84)	1568 (8.47)	5362 (7.66)	
Non‐Hispanic Black	8373 (11.43)	1806 (11.51)	6567 (11.41)	
Non‐Hispanic White	17,261 (69.00)	3439 (67.67)	13,822 (69.39)	
Other Race	6168 (11.73)	1498 (12.35)	4670 (11.54)	
Education level (*n*, %)				< 0.001
Below high school	10,907 (18.33)	1726 (13.40)	9181 (19.78)	
High school graduate or GED	9222 (25.10)	1827 (22.25)	7395 (25.94)	
Some college or above	18,603 (56.57)	4758 (64.36)	13,845 (54.29)	
Marital status (*n*, %)				< 0.001
Married or living with a partner	22,809 (62.42)	4635 (60.49)	18,174 (62.99)	
Never married	6536 (16.13)	2594 (26.83)	3942 (13.01)	
Widowed, divorced, or separated	9387 (21.44)	1082 (12.68)	8305 (24.01)	
Smoking status (*n*, %)				< 0.001
Former	10,163 (26.81)	1409 (19.80)	8754 (28.86)	
Never	20,470 (51.93)	5023 (58.65)	15,447 (49.96)	
Now	8099 (21.27)	1879 (21.55)	6220 (21.18)	
Alcohol use (*n*, %)				< 0.001
Active alcohol user	25,225 (71.24)	6273 (79.39)	18,952 (68.86)	
Nonactive alcohol user	13,507 (28.76)	2038 (20.61)	11,469 (31.14)	
Poverty‐to‐income ratio	2.92 ± 0.03	3.02 ± 0.04	2.88 ± 0.03	< 0.001
Physical activity total METs/week	3175.73 ± 51.40	3651.02 ± 108.50	3036.62 ± 51.11	< 0.001
eGFR, mL/min/1.73 m²	91.80 ± 0.27	103.08 ± 0.38	88.50 ± 0.28	< 0.001
Fasting plasma glucose, mg/dL	108.53 ± 0.28	94.44 ± 0.18	112.65 ± 0.34	< 0.001
HbA1c (%)	5.69 ± 0.01	5.24 ± 0.01	5.82 ± 0.01	< 0.001
Albumin, g/dL	42.60 ± 0.04	43.31 ± 0.06	42.39 ± 0.05	< 0.001
Serum creatinine, mg/dL	0.90 ± 0.00	0.83 ± 0.00	0.92 ± 0.00	< 0.001
Triglycerides, mg/dL	182.73 ± 1.33	79.46 ± 0.42	212.95 ± 1.58	< 0.001
Total cholesterol, mg/dL	197.43 ± 0.43	184.02 ± 0.68	201.36 ± 0.47	< 0.001
LDL‐C, mg/dL	109.19 ± 0.33	110.04 ± 0.55	108.94 ± 0.37	0.08
HDL‐C, mg/dL	52.13 ± 0.17	58.10 ± 0.26	50.38 ± 0.17	< 0.001
Body mass index, kg/m²	29.51 ± 0.07	25.98 ± 0.09	30.54 ± 0.08	< 0.001
Waist circumference, cm	100.72 ± 0.18	90.08 ± 0.22	103.83 ± 0.18	< 0.001
DII	1.46 ± 0.02	1.30 ± 0.04	1.51 ± 0.02	< 0.001
Hypertension (*n*, %)				< 0.001
No	18,308 (49.34)	8311 (100.00)	9997 (34.51)	
Yes	20,424 (50.66)	0 (0.00)	20,424 (65.49)	
Diabetes mellitus (*n*, %)				< 0.001
No	30,323 (82.42)	8311 (100.00)	22,012 (77.28)	
Yes	8409 (17.58)	0 (0.00)	8409 (22.72)	
Coronary heart disease (*n*, %)				< 0.001
No	36,722 (95.31)	8255 (99.33)	28467 (94.13)	
Yes	2010 (4.69)	56 (0.67)	1954 (5.87)	
Congestive heart failure (*n*, %)				< 0.001
No	37,153 (96.77)	8276 (99.60)	28,877 (95.93)	
Yes	1579 (3.23)	35 (0.40)	1544 (4.07)	
Stroke (*n*, %)				< 0.001
No	36,894 (96.18)	8242 (99.11)	28,652 (95.32)	
Yes	1838 (3.82)	69 (0.89)	1769 (4.68)	
Chronic kidney disease (*n*, %)				< 0.001
No	29,199 (79.36)	8311 (100.00)	20,888 (73.32)	
Yes	9533 (20.64)	0 (0.00)	9533 (26.68)	
Metabolic syndrome (*n*, %)				< 0.001
No	23,290 (59.71)	8311 (100.00)	14,979 (47.92)	
Yes	15,442 (40.29)	0 (0.00)	15,442 (52.08)	
All‐cause mortality (*n*, %)				< 0.001
No	31,714 (85.80)	7939 (96.17)	23,775 (82.76)	
Yes	7018 (14.20)	372 (3.83)	6646 (17.24)	

*Note:* Continuous variables are expressed as the mean ± standard deviation (SD), while categorical variables are reported as frequencies with corresponding percentages.

Abbreviations: DII, dietary inflammatory index; eGFR, estimated glomerular filtration rate; GED, general educational development; HbA1c, hemoglobin A1c; HDL‐C, high‐density lipoprotein cholesterol; IH–CKM, intermediate‐to‐high‐risk cardiovascular‐kidney‐metabolic syndrome; LDL‐C, low‐density lipoprotein cholesterol; MET, metabolic equivalent of task.

### 3.2 Relationship Between DII Score and IH–CKM Score

**Figure 2:**
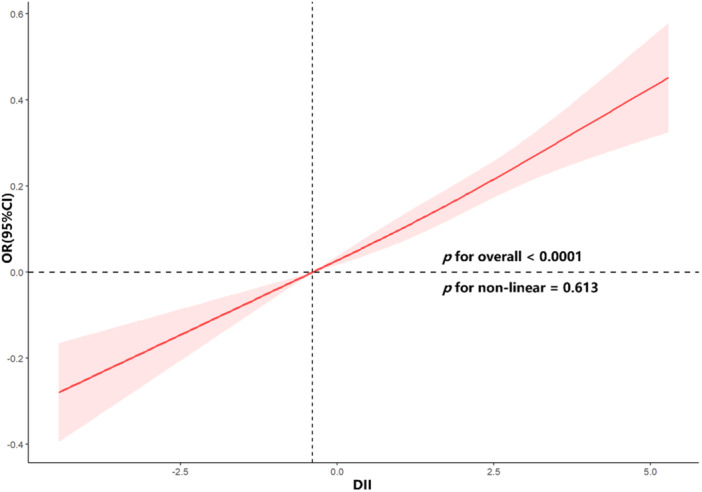
Restricted cubic spline curve illustrating the association between DII and IH–CKM.

Weighted logistic regression analysis was conducted to assess the relationship between DII score and risk of IH–CKM (Table 2). In the unadjusted model (Model 1), a significant positive association was observed between DII and IH–CKM (OR: 1.06, 95% CI: 1.04–1.09, *p* < 0.001). This association remained significant after adjusting for age, sex, and race in Model 2 (OR: 1.11, 95% CI: 1.08–1.13, *p* < 0.001) and persisted even after full adjustment for all covariates in Model 3 (OR: 1.08, 95% CI: 1.05–1.10, *p* < 0.001). When DII was analyzed by quartile, higher DII scores (Q2, Q3, and Q4) were associated with an increased likelihood of IH–CKM compared with the reference quartile (Q1). In the fully adjusted model (Model 3), the ORs for Q2, Q3, and Q4 were 1.17 (95% CI: 1.05–1.31, *p* = 0.01), 1.32 (95% CI: 1.19–1.47, *p* < 0.001), and 1.44 (95% CI: 1.27–1.63, *p* < 0.001), respectively, indicating a significant trend across quartiles (P for trend < 0.001). A dose–response relationship between DII and risk of IH–CKM was further evaluated via restricted cubic spline models, which revealed a significant positive linear association (nonlinearity *p* = 0.613; Figure 2).

**Table 2:** ORs (95% CIs) for IH–CKM according to DII.

Characteristic	Model 1		Model 2		Model 3	
	OR (95% CI)	*p*	OR (95% CI)	*p*	OR (95% CI)	*p*
DII	1.06 (1.04, 1.09)	< 0.001	1.11 (1.08, 1.13)	< 0.001	1.08 (1.05, 1.10)	< 0.001
Q1	Reference	—	Reference	—	Reference	—
Q2	1.14 (1.03, 1.26)	0.01	1.22 (1.09, 1.36)	< 0.001	1.17 (1.05, 1.31)	0.01
Q3	1.25 (1.13, 1.39)	< 0.001	1.42 (1.28, 1.59)	< 0.001	1.32 (1.19, 1.47)	< 0.001
Q4	1.34 (1.19, 1.50)	< 0.001	1.63 (1.44, 1.84)	< 0.001	1.44 (1.27, 1.63)	< 0.001
*p* for trend		< 0.001		<0.001		< 0.001

*Note:* Model 1: Unadjusted.

Model 2: Adjusted for age, sex, and race.

Model 3: Adjusted for age, sex, race, education level, marital status, Poverty‐to‐income ratio, smoking status, alcohol use, physical activity.

Abbreviations: CI, confidence interval; DII, dietary inflammatory index; IH–CKM, intermediate‐to‐high‐risk cardiovascular‐kidney‐metabolic syndrome; OR, odds ratio.

**Figure 3:**
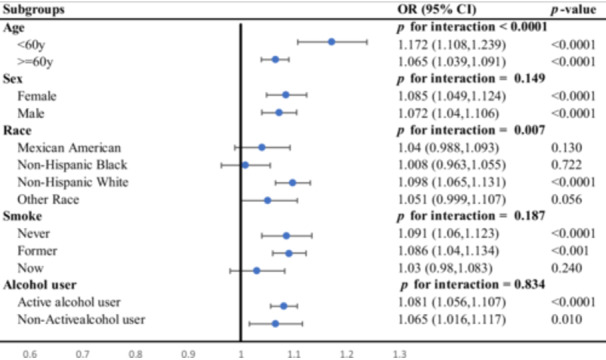
Subgroup analyses assessing the robustness of the association between dietary inflammatory index (DII) and IH–CKM.

To assess the robustness of the main findings, prespecified subgroup analyses stratified by age, sex, race/ethnicity, smoking status, and alcohol consumption were conducted (Figure 3). The positive association between DII and IH–CKM was significant across most subgroups, with notable interactions observed for age and race (*P* values for interactions < 0.001 and 0.007, respectively). Specifically, for participants under 60 years old, each one‐unit increase in DII was associated with a 17.2% increase in odds of IH–CKM (OR: 1.172, 95% CI: 1.108–1.239, *p* < 0.001), whereas for those aged 60 and above, the increase was 6.5% (OR: 1.065, 95% CI: 1.039–1.091, *p* < 0.001). Racial differences were also observed, with a significant association in non‐Hispanic whites (OR: 1.098, 95% CI: 1.065–1.131, *p* < 0.001) but not in other racial groups. Overall, missingness across covariates was low to moderate, with no variable exhibiting more than approximately 10% missing data.

### 3.3 Correlation Between DII and All‐Cause Mortality

**Figure 4:**
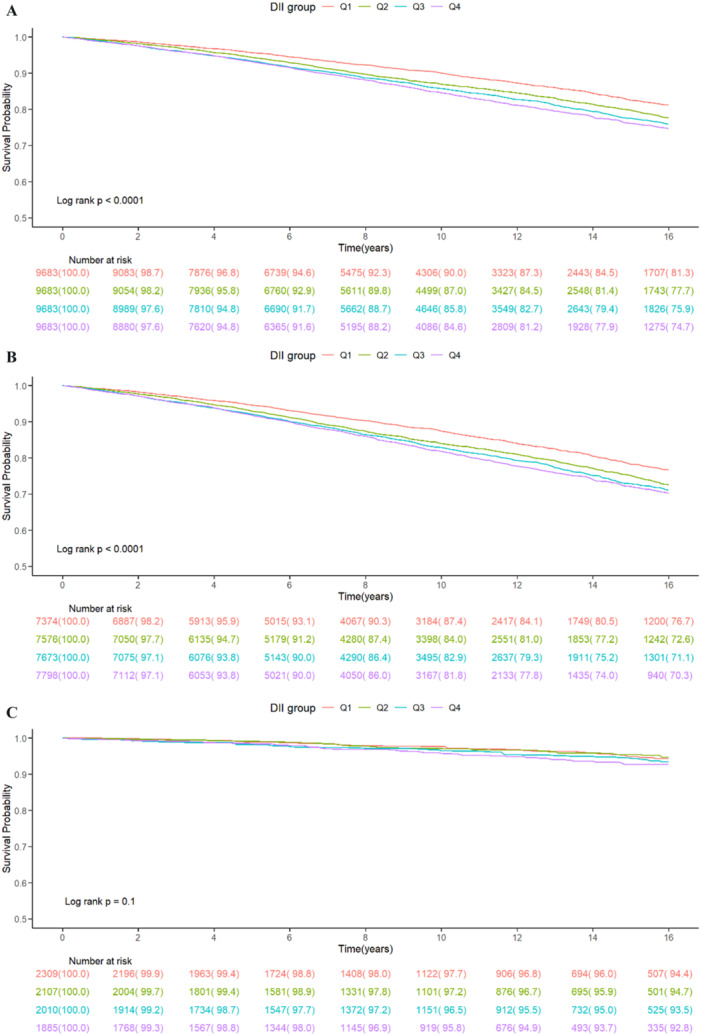
Kaplan–Meier analysis of all‐cause mortality for (A) total participants, (B) IH–CKM participants, and (C) non‐IH–CKM participants.

Among all participants, 7018 (14.2%) deaths occurred. Kaplan–Meier survival curves demonstrated that higher DIIs were associated with an increased risk of all‐cause mortality in both the overall population and IH–CKM participants (log‐rank *p* < 0.001, Figure 4A,B). However, this association was not statistically significant in the non‐IH–CKM group (log‐rank *p* = 0.1, Figure 4C).

According to the multivariate Cox regression analysis (Table 3), each one‐unit increase in DII (range: −5.281 to 5.795) was associated with an adjusted hazard ratio (HR) of 1.05 (95% CI: 1.03–1.07, *p* < 0.001) for all participants, 1.04 (95% CI: 1.02–1.06, *p* < 0.001) for IH–CKM participants, and 1.15 (95% CI: 1.06–1.26, *p* = 0.001) for non‐IH–CKM participants after adjusting for all confounding factors. Although the Kaplan–Meier comparison in the non‐IH‐CKM group did not reach statistical significance (log‐rank *p* = 0.10), the multivariable Cox proportional hazards model demonstrated a significant association between DII and all‐cause mortality (HR = 1.15, *p* = 0.001). This discrepancy likely reflects methodological differences, as Kaplan–Meier curves are unadjusted and may have reduced statistical power with fewer events, whereas Cox models account for confounding variables and utilize time‐to‐event information more efficiently.

**Table 3:** HRs (95% CIs) for all‐cause mortality according to DII.

Characteristic	Model 1		Model 2		Model 3	
	HR (95% CI)	*p*	HR (95% CI)	*p*	HR (95% CI)	*p*
All participants						
DII	1.08 (1.06, 1.10)	< 0.001	1.11 (1.09, 1.13)	< 0.001	1.05 (1.03, 1.07)	< 0.001
Q1	Reference	—	Reference	—	Reference	—
Q2	1.23 (1.11, 1.36)	< 0.001	1.27 (1.16, 1.39)	< 0.001	1.16 (1.05, 1.28)	0.002
Q3	1.36 (1.24, 1.49)	< 0.001	1.46 (1.34, 1.61)	< 0.001	1.21 (1.10, 1.34)	< 0.001
Q4	1.47 (1.34, 1.61)	< 0.001	1.61 (1.47, 1.75)	< 0.001	1.22 (1.11, 1.34)	< 0.001
*p* for trend		< 0.001		< 0.001		< 0.001
IH–CKM						
DII	1.07 (1.05, 1.09)	< 0.001	1.10 (1.08, 1.12)	< 0.001	1.04 (1.02, 1.06)	< 0.001
Q1	Reference	—	Reference	—	Reference	—
Q2	1.22 (1.10, 1.35)	< 0.001	1.25 (1.13, 1.38)	< 0.001	1.15 (1.04, 1.27)	0.01
Q3	1.31 (1.20, 1.44)	< 0.001	1.41 (1.29, 1.54)	< 0.001	1.18 (1.07, 1.30)	< 0.001
Q4	1.39 (1.26, 1.54)	< 0.001	1.52 (1.38, 1.67)	< 0.001	1.17 (1.07, 1.29)	0.001
*p* for trend		< 0.001		< 0.001		0.001
Non‐IH–CKM						
DII	1.09 (1.02, 1.16)	0.01	1.25 (1.16, 1.34)	< 0.001	1.15 (1.06, 1.26)	0.001
Q1	Reference	—	Reference	—	Reference	—
Q2	0.96 (0.63, 1.46)	0.84	1.19 (0.78, 1.83)	0.42	1.08 (0.68, 1.70)	0.75
Q3	1.32 (0.90, 1.93)	0.16	1.99 (1.30, 3.06)	0.002	1.57 (1.02, 2.43)	0.04
Q4	1.40 (0.98, 2.00)	0.07	2.60 (1.79, 3.80)	< 0.001	1.78 (1.10, 2.86)	0.02
*p* for trend		0.019		< 0.001		0.005

*Note:* Model 1: Unadjusted.

Model 2: Adjusted for age, sex, and race.

Model 3: Adjusted for age, sex, race, education level, marital status, Poverty‐to‐income ratio, smoking status, alcohol use, physical activity

Abbreviations: CI, confidence interval; DII, dietary inflammatory index; HR, hazard ratio; IH–CKM, intermediate‐to‐high‐risk cardiovascular‐kidney‐metabolic syndrome.

**Figure 5:**
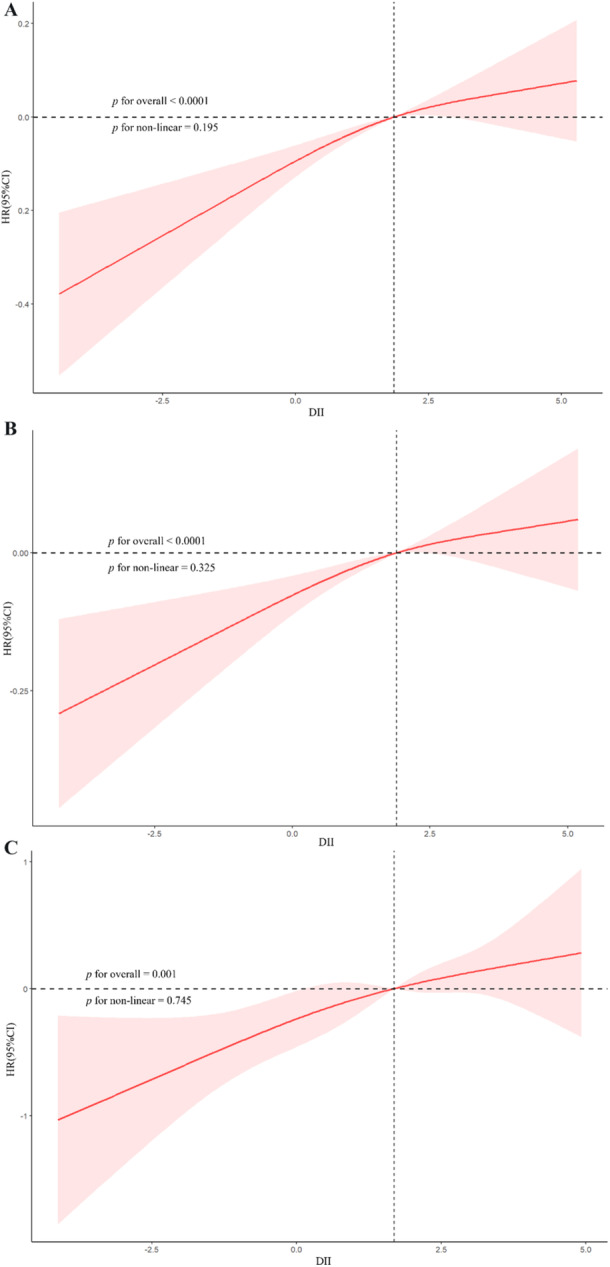
Restricted cubic spline curves illustrating the association between the DII and all‐cause mortality in (A) total participants, (B) IH–CKM participants, and (C) non‐IH–CKM participants.

When DII was analyzed as a categorical variable, participants in the highest quartile (Q4) had a significantly greater risk of mortality than those in the lowest quartile (Q1), with adjusted HRs of 1.22 (95% CI: 1.11–1.34; *p* < 0.001) in the overall population, 1.17 (95% CI: 1.07–1.29; *p* = 0.001) in the IH–CKM group, and 1.78 (95% CI: 1.10–2.86; *p* = 0.02) in the non‐IH–CKM group. A significant trend was observed across all quartiles in each model (*p* for trend < 0.05). Notably, the hazard ratio for participants in the highest DII quartile was accompanied by a relatively wide confidence interval, likely reflecting the limited number of death events in this subgroup; therefore, these estimates should be interpreted with caution. The RCS analysis further evaluated the relationship between DII and all‐cause mortality (Figure 5), revealing a linear positive association between DII and adjusted mortality risk for all participants (nonlinearity *p* = 0.195), IH–CKM participants (nonlinearity *p* = 0.325), and non‐IH–CKM participants (nonlinearity *p* = 0.745).

## 4 Discussion

This study examined the association between the DII and both intermediate‐to‐high‐risk cardiovascular–kidney–metabolic syndrome (IH–CKM) and all‐cause mortality in a nationally representative U.S. cohort. We found that higher DII scores, reflecting diets with greater inflammatory potential, were associated with increased risk of IH–CKM. A dose–response relationship was observed after multivariable adjustment, indicating that dietary inflammatory burden may play a contributory role in cardiometabolic risk accumulation. In addition, elevated DII was associated with higher all‐cause mortality among individuals both with and without IH–CKM, underscoring the potential relevance of dietary inflammation across different metabolic phenotypes.

The observed association between proinflammatory dietary patterns and IH–CKM is consistent with, and extends, findings from recent population‐based studies examining dietary inflammation and cardiometabolic risk. A large contemporary analysis published in *Nutrients* demonstrated that higher DII scores were associated with adverse cardiometabolic profiles and increased risk of chronic disease across multiple metabolic domains, underscoring the central role of diet‐induced inflammation in systemic metabolic dysregulation [29]. Previous studies have implicated dietary‐induced inflammation in the development and progression of CKM‐related conditions [30]. Tain and Hsu reported that greater inflammatory load in maternal diets was associated with unfavorable CKM profiles in offspring, emphasizing the long‐term implications of early‐life dietary exposures [1]. Similarly, Al‐Chalabi et al. found that individuals with obesity—often characterized by elevated DII—exhibited a higher propensity for developing cardio‐renal‐metabolic abnormalities, reinforcing the relevance of diet‐derived inflammation in CKM pathogenesis [31]. In addition to dietary indices, systemic inflammatory markers have also been correlated with CKM features. Gao et al. demonstrated a significant positive association between the SII and CKM syndrome, highlighting inflammation as a central mechanism connecting metabolic and cardiovascular dysfunction [11]. Guldan et al. further expanded this perspective by identifying interactions between dietary inflammation and hormonal pathways, proposing that endocrine responses may amplify the deleterious impact of proinflammatory diets on IH–CKM development [32]. Conversely, in their exploration of maternal polyphenol intake, Tain and Hsu observed that diets enriched with anti‐inflammatory polyphenols were inversely associated with CKM‐related health issues, lending support to the protective role of anti‐inflammatory dietary patterns [33]. Collectively, these findings corroborate our results and support the hypothesis that higher DII scores are linked to a greater risk of IH–CKM and elevated mortality. The evidence underscores the importance of dietary modulation as a potential intervention strategy to mitigate inflammation‐driven metabolic and cardiovascular deterioration.

From a pathophysiological perspective, the observed associations are biologically plausible within the CKM continuum. Pro‐inflammatory dietary patterns characterized by high DII scores have been shown to promote chronic low‐grade systemic inflammation, which serves as a central driver of endothelial dysfunction, oxidative stress, and vascular remodeling. Recent experimental and clinical evidence indicates that inflammatory diets can impair endothelial nitric oxide bioavailability and accelerate atherosclerotic processes, thereby increasing cardiovascular vulnerability [34]. In parallel, emerging research highlights the critical role of the gut microbiota–inflammation axis in mediating diet‐related cardiometabolic injury. Diets with high inflammatory potential can alter gut microbial composition, increase intestinal permeability, and facilitate translocation of endotoxins, which in turn amplify systemic inflammation and metabolic dysfunction [35]. These inflammatory cascades may accelerate renal interstitial fibrosis and maladaptive cardiac remodeling, including left ventricular hypertrophy, thereby contributing to CKM progression. Collectively, these interrelated inflammatory and vascular processes provide a coherent mechanistic explanation for the link between proinflammatory dietary patterns and elevated IH–CKM risk observed in this study.

Multiple epidemiological investigations have consistently demonstrated that elevated DII scores are linked to an increased risk of all‐cause mortality. In both the SUN and PREDIMED cohorts, Garcia‐Arellano et al. found that individuals with more proinflammatory diets exhibited significantly higher mortality rates, particularly from cardiovascular and oncological causes [36]. Similarly, an analysis by Cao et al. using NHANES data indicated that older hypertensive adults with higher DII values had markedly increased all‐cause mortality risk [37]. In a prospective cohort study, Shivappa et al. reported a robust association between elevated DII scores and mortality, even after controlling for a comprehensive set of lifestyle and health‐related confounders [38]. Park et al. further validated this relationship in a nationally representative U.S. adult population, highlighting that dietary inflammation was independently associated with increased mortality risk across multiple causes [39]. Supporting evidence from non‐Western populations also exists. In a large‐scale Japanese cohort, Okada et al. observed that individuals with higher DII scores experienced significantly elevated risks of all‐cause mortality, suggesting that the detrimental effects of proinflammatory diets extend across ethnic boundaries [40]. Moreover, findings from the Melbourne Collaborative Cohort Study by Hodge et al. revealed a positive association between DII and both total and cardiovascular‐specific mortality, further reinforcing the role of dietary inflammation in influencing long‐term health outcomes [41]. Taken together, these studies provide converging evidence that proinflammatory dietary patterns, as captured by higher DII scores, are consistently associated with increased all‐cause mortality. This relationship has been demonstrated across diverse populations, geographic regions, and study designs, underscoring the global relevance of diet‐related inflammation in mortality risk assessment.

The fact that a significant DII–IH‐CKM association emerged only among non‐Hispanic Whites is likely multifactorial. First, this group represented nearly 70% of our analytic sample, whereas Mexican Americans, non‐Hispanic Blacks, and participants of other races accounted for markedly smaller proportions (7.84%, 11.43%, and 11.73%, respectively). The resulting disparity in statistical power may have obscured weaker associations in the smaller subgroups.

Dietary culture also differs substantially by ethnicity and can shift the inflammatory load of habitual eating patterns. Populations adhering more closely to Mediterranean‐type diets—characterized by high intakes of fiber, antioxidants, and unsaturated fats—tend to exhibit lower systemic inflammation and reduced cardiometabolic risk [42]. Conversely, Western dietary patterns rich in saturated fat, refined carbohydrate, and ultra‐processed foods elevate inflammatory biomarkers and are linked to higher DII values [21, 43]. Socio‐economic context further modifies diet quality. Limited financial resources and restricted access to fresh foods predispose lower‐income communities to pro‐inflammatory eating habits, thereby amplifying metabolic and inflammatory disease burdens [44, 45]. Finally, gene–diet interactions may differ across ancestral backgrounds. Variants in FADS1/2, which regulate long‐chain PUFA metabolism, can modulate the anti‐inflammatory effects of ω‐3 fatty acids [46, 47]. Likewise, APOE polymorphisms influence lipid and inflammatory responses to dietary fat; certain APOE genotypes intensify inflammation in the setting of high‐fat diets, potentially aggravating CKM risk [48]. Taken together, the blend of sample‐size constraints, culturally patterned diets, socio‐economic food environments, and ancestry‐dependent genetic architecture provides a plausible explanation for why the DII signal reached statistical significance only in non‐Hispanic Whites in our analysis.

Elevated DII scores may contribute to the development of IH–CKM and excess mortality through inflammatory mechanisms that directly affect both renal and cardiovascular structure and function. High‐DII diets are enriched in proinflammatory nutrients that upregulate cytokines such as IL‐6, TNF‐α, and CRP, thereby promoting chronic low‐grade systemic inflammation [49]. Sustained inflammatory activation may accelerate renal interstitial fibrosis via cytokine‐mediated pathways, contributing to progressive kidney dysfunction—one of the defining features of IH–CKM [50]. At the cardiovascular level, systemic inflammation promotes endothelial dysfunction, oxidative stress, and maladaptive cardiac remodeling, including left ventricular hypertrophy, which increases long‐term cardiovascular risk within the CKM spectrum [51]. Collectively, these interrelated inflammatory and vascular processes provide a plausible mechanistic link between proinflammatory dietary patterns and CKM progression.

Notably, the association between dietary inflammatory burden and all‐cause mortality was more pronounced among participants without established intermediate‐to‐high‐risk CKM. This pattern has also been reported in recent population‐based studies, suggesting that dietary inflammation may exert deleterious metabolic effects at early, preclinical stages of cardiometabolic disease. A recent cohort analysis published in Diabetes Research and Clinical Practice found that higher DII scores were significantly associated with insulin resistance, dysglycemia, and increased mortality risk even among individuals without diagnosed cardiometabolic conditions [52]. Together, these findings indicate that pro‐inflammatory dietary patterns may function as upstream risk factors, contributing to cumulative metabolic and inflammatory burden before overt CKM develops, underscoring the potential value of early dietary and inflammation‐targeted prevention strategies.

This study offers several noteworthy strengths, particularly in its novelty and integrative design. To our knowledge, it is the first to comprehensively investigate the association between the DII, IH–CKM (stages 2–4), and all‐cause mortality using both cross‐sectional and longitudinal frameworks. Unlike prior research that predominantly examined isolated CKM components, our analysis focuses specifically on IH–CKM as a composite outcome, thereby providing a more holistic understanding of inflammation‐related cardiometabolic risk. Nonetheless, certain limitations must be acknowledged. First, dietary intake data were based on self‐reported 24‐h recalls, which are inherently susceptible to recall bias and inaccuracies in estimating portion sizes or consumption frequency [53]. Future work may benefit from incorporating objective dietary assessment tools—such as nutritional biomarkers or digital tracking technologies—to enhance measurement precision [54]. Second, despite adjustment for a broad range of demographic, socioeconomic, and lifestyle factors, residual confounding cannot be fully excluded. The use of medications with potential anti‐inflammatory effects, including nonsteroidal anti‐inflammatory drugs, statins, or antidiabetic agents, was not explicitly accounted for and may influence both inflammatory burden and CKM progression. In addition, although PIR and education were included as socioeconomic proxies, other social determinants—such as healthcare access, food security, and neighborhood nutrition environments—were not captured, potentially contributing to residual social gradient bias. Third, the observational design precludes causal inference, and the lack of longitudinal dietary data limits evaluation of temporal changes in DII and their influence on CKM stage progression. In addition, future studies incorporating repeated dietary assessments together with biomarker‐based inflammatory measurements will be required to better characterize the temporal relationship between dietary inflammation and CKM progression. Fourth, limitations related to CKM classification should be acknowledged. CKM stage 3 was partly defined using predicted cardiovascular risk rather than direct subclinical biomarkers, which are unavailable in NHANES, while CKM stage 4 relied on self‐reported cardiovascular disease, potentially introducing misclassification. Moreover, etiological heterogeneity within IH–CKM stages, such as diabetic versus hypertensive nephropathy, could not be differentiated. Fifth, survival analyses focused on all‐cause mortality and did not account for competing risks. In high‐risk IH–CKM populations, competing events such as renal failure or cancer‐related death may precede cardiovascular mortality, potentially leading to underestimation of diet‐related cardiovascular risk. Additional constraints include top‐coding of age ≥ 80 years in NHANES, which may underestimate risk among the oldest participants, and inherent limitations of the DII. Specifically, DII weights were derived largely from earlier literature and may not capture recently recognized anti‐inflammatory components, including gut microbiota–derived metabolites. The use of global reference intake values may further limit comparability with U.S. population–specific inflammatory baselines. Finally, the NHANES cycles analyzed were collected more than a decade ago. Although suitable for etiological inference, temporal changes in dietary patterns and CKM risk profiles may limit generalizability to contemporary populations, highlighting the need for validation in more recent cohorts.

The observed associations between DII and both IH–CKM risk and all‐cause mortality have meaningful implications for clinical and public health strategies. From a translational perspective, our findings suggest that diet‐related inflammation represents a potentially modifiable target for early CKM risk mitigation. In clinical settings, individuals with proinflammatory dietary patterns may benefit from targeted nutritional counseling aimed at reducing systemic inflammation, such as increasing intake of anti‐inflammatory foods—including fiber‐rich vegetables, fruits, and unsaturated fats—while limiting refined carbohydrates and saturated fats. Importantly, these dietary strategies are generally aligned with existing nutritional recommendations and can be implemented without specialized resources, supporting their practical feasibility. Although comprehensive DII calculation requires detailed dietary data, simplified dietary screening approaches or abbreviated indices focusing on key inflammatory components may facilitate integration into routine care and population‐level prevention programs. Future research should focus on validating such streamlined tools and evaluating their utility in supporting inflammation‐targeted dietary interventions. At the population level, promoting anti‐inflammatory eating patterns may contribute to reducing IH–CKM burden and related morbidity and mortality, reinforcing dietary inflammation control as a public health priority.

## 5 Conclusion

DII score was positively associated with a higher risk of IH–CKM. In addition, higher DII scores were linked to increased all‐cause mortality both among individuals with and without IH–CKM. From a clinical perspective, DII may serve as a useful indicator for identifying individuals at elevated risk of diet‐related inflammation.

## Author Contributions

H.L., M.L., and Y.Y. contributed equally to the conceptualization and design of the study. M.L., Y.Z., and S.C. performed the formal statistical analyses. Data curation was conducted by L.W., Y.W., J.L., M.L., Y.Z., and S.C. Visualization and figure preparation were carried out by C.G. The original draft of the manuscript was written by H.L. and M.L. All authors participated in the critical review, editing, and revision of the manuscript. C.Z. supervised the study, oversaw project administration, and takes full responsibility for the integrity of the data and the accuracy of the analysis. All authors read and approved the final manuscript.

## Policy on Using ChatGPT and Similar AI Tools

The authors confirm that no AI or AI‐assisted technologies were used during the study design, data analysis, manuscript writing, or editing process.

## Disclosure

The lead author Chuanwei Zhao affirms that this manuscript is an honest, accurate, and transparent account of the study being reported; that no important aspects of the study have been omitted; and that any discrepancies from the study as planned (and, if relevant, registered) have been explained.

## Ethics Statement

The NHANES study protocol was approved by the NCHS Research Ethics Review Committee and conducted under the oversight of the Centers for Disease Control and Prevention (CDC) and NCHS. Informed written consent was obtained from all participants.

## Consent

All authors have read and approved the final version of the manuscript. The corresponding author, Chuanwei Zhao, had full access to all of the data in this study and takes complete responsibility for the integrity of the data and the accuracy of the data analysis.

## Conflicts of Interest

The authors declare no conflicts of interest.

## Data Availability

The data analyzed in this study are publicly available from the NHANES database on the Centers for Disease Control and Prevention (CDC) website (https://www.cdc.gov/nchs/nhanes/). All data supporting the findings of this work are contained within the article and its supporting materials. NHANES data are fully de‐identified; therefore, no additional ethical approval was required for this secondary analysis.
